# The influence of electrical effects on device performance of organic solar cells with nano-structured electrodes

**DOI:** 10.1038/s41598-017-05591-8

**Published:** 2017-07-13

**Authors:** Mina Mirsafaei, Amir Hossein Fallahpour, Paolo Lugli, Horst-Günter Rubahn, Jost Adam, Morten Madsen

**Affiliations:** 1 SDU NanoSYD, Mads Clausen Institute, University of Southern Denmark, Alsion 2, Sønderborg, DK-6400 Denmark; 2 Department of Electrical and Computer Engineering, Institute for Nanoelectronics, Technical University of Munich, Arcisstr, 21 80333 Munich Germany

## Abstract

Integration of light-trapping features and exploitation of metal nanostructure plasmonic effects are promising approaches for enhancing the power conversion efficiency of organic solar cells. These approaches’ effects on the light absorption enhancement have been widely studied, especially in inorganic devices. While this light-trapping concept can be transferred to organic devices, one has to also consider nanostructure-induced electrical effects on the device performance, due to the fundamental difference in the organic semiconducting material properties compared to their inorganic counterparts. In this contribution, we exemplarily model the electrical properties of organic solar cells with rectangular-grating structures, as compared to planar reference devices. Based on our numeric results, we demonstrate that, beyond an optical absorption enhancement, the device fill factor improves significantly by introducing the grating structures. From the simulations we conclude that enhanced carrier collection efficiency is the main reason for the increased solar cell fill factor. This work contributes towards a more fundamental understanding of the effect of nanostructured electrodes on the electrical properties of organic solar cells.

## Introduction

Low material and fabrication cost, the ease of processing organic materials and the possibility of making ultra-thin, flexible cells using techniques, such as solution processes^1, 2^, printing^3, 4^ and roll-to-roll technology^5^, render organic solar cells (OSCs) ideal candidates for the future renewable energy market. The organic material properties, such as the molecular mass^6^, the band gap and absorption properties^7, 8^, can easily be tailored through modifying the length and presence of different functional groups^9^, which makes them interesting for a wide range of opto-electronic applications. In recent years, performance improvements of organic solar cells have led to high power conversion efficiencies (PCE)^10–13^, currently having a record of 13.2%, set by Heliatek. Nevertheless, organic photoactive materials come with certain inherent drawbacks such as a relatively narrow absorption band, short exciton diffusion lengths and low charge carrier mobility^14^.

To meet these drawbacks and to further increase the device efficiency, various light management techniques have been investigated to achieve optical field enhancement in the photoactive layer region. These approaches are based on plasmonic, diffraction and scattering effects, e.g., introduced by the integration of metallic nanoparticles near or in the photoactive layer, by the inclusion of metallic and non-metallic, aperiodic or (quasi-) periodic surface structures, or by the integration of randomly distributed metallic nanoparticles in the back contact layer^15–17^. There are several reports on nanostructured inorganic solar cell efficiency improvements as a result of enhanced optical absorption, which is a dominant factor to increase the overall performance and, in this case, directly translates to an increased photocurrent^18^. Less attention has been paid to the electrical effect from incorporation of plasmonic nanostructures in inorganic solar cells, due to the large photoactive layer thickness, very high charge carrier mobility, and efficient charge collection efficiency. It has however recently been demonstrated that nanostructures can lead to improved electrical properties in inorganic solar cells, focusing mainly on the improved electrical contacting arising from the different nanostructures^19^. In organic devices, due to the fundamental differences between the opto-electronic properties of the organic semiconductors and inorganic materials, it becomes necessary to also consider improvements coming from the electrical properties introduced by nanostructures, alongside the optical absorption enhancement^20–22^.

Although the electrical processes taking place in organic solar cells, e.g., the exciton generation/dissociation, charge carrier transport, recombination loss mechanisms, and interfacial morphology effects, have been experimentally investigated for nanostructured OSCs^23–27^, a theoretical/numerical study for a comprehensive prediction of electrical effects arising from the integration of such complex non-planar nanostructures is, to date, still missing.

The integrated light-trapping nanostructures are likely to affect physical processes such as charge carrier losses, transport and collection efficiency. These processes depend on several factors, such as the electric field distribution, the exciton separation process and the spatial concentration dependence of carrier recombination, effectively influencing the final PCE of the cell.

To address the issue of tracing the electrical device properties more accurately, we perform numerical simulations of nanostructured organic solar cells, considering the involved underlying organic semiconductor physics. We compute the optical and electrical device properties, using three-dimensional (3D) finite-difference time-domain (FDTD) and finite element (FE) methods, respectively. In particular, we investigate the contact behavior, the exciton dynamics, and the charge carrier transport for disordered materials. Building on that, as a case study, we perform full electrical simulations of a well-known conventional organic solar cell setup with a back contact grating structure, to understand how nanostructuring influences the electrical properties of such organic devices. As a result, we find a significant improvement of the electrical properties in the investigated nanostructured organic solar cell, beyond what is governed by the optical absorption enhancements, originating merely from the implemented grating structure.

## Results and Discussion

**Figure 1:**
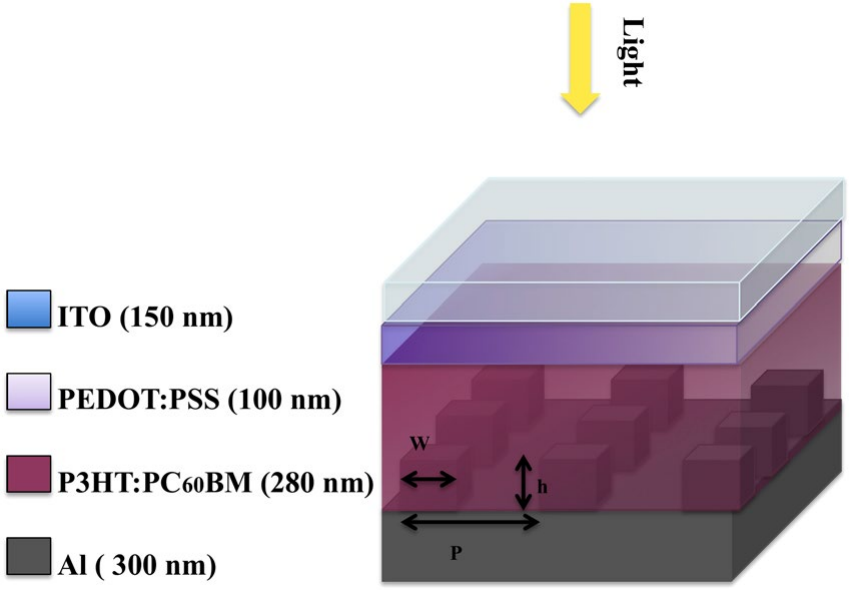
Organic solar cell model geometry, with a back contact grating and material thicknesses as used in the simulations (h – pillar height [nm], p – grating period [nm], w – pillar width [nm]).

The exemplary chosen conventional OSC structure studied in this work is composed of the following well-known multilayer structure (Fig. 1): Indium thin oxide (ITO)/poly-3,4-ethylenedioxythiophene:poly(styrenesulfonate) (PEDOT:PSS) /Poly(3-hexylthiophene-2,5-diyl)(P3HT) blended with [6,6]-phenyl C61 butyric acid methyl ester(PC_60_BM)/Aluminum(Al).

**Figure 2:**
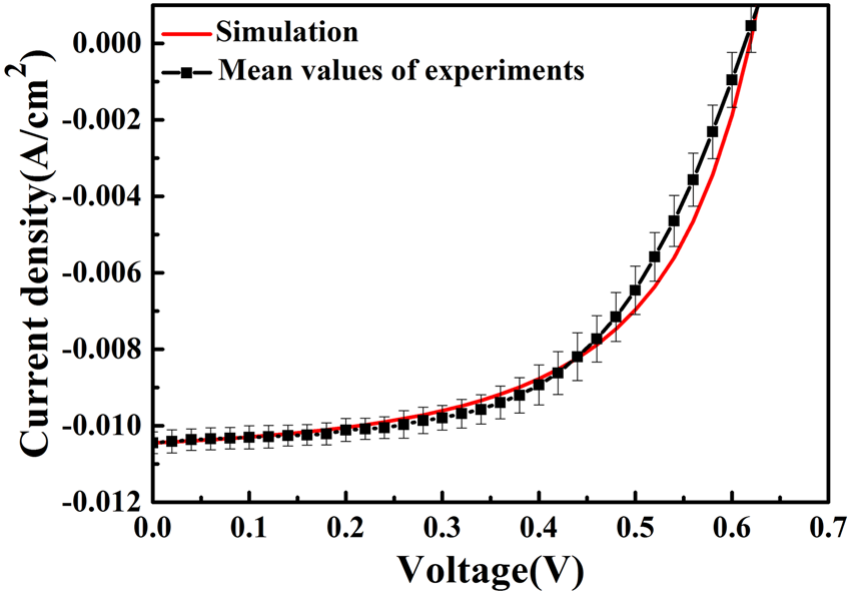
Experimental^28^ and simulated results for current density versus voltage curves for a planar organic solar cell with a 200 nm thick active layer. Our model widely agrees with the experimental results.

In our study, in order to first investigate simulation parameter consistency and to verify our electrical model, we fitted the model to experimentally measured data for a planar reference device structure^28^. All electrical parameters used for the fitting are directly taken from the literature or estimated by experimental measurements (Table 1). Figure 2 demonstrates the established agreement between the experimental and simulated results for current density versus voltage (*J*-*V*) curves for a planar organic solar cell with a 200 nm thick active layer.

**Table 1:** Electrical simulation parameters.

Description	Parameter	Value	Ref.
Photoactive layer thickness	*L*	200 nm	
Cathode (Al) work function	Φ_cathode_	− 4.1	42
Anode (PEDOT) work function	Φ_anode_	−5.2	43
HOMO level onset	*E*_HOMO_	−5.1 eV	44–46
LUMO level onset	*E*_LUMO_	−3.9 eV	44–46
Decay rate of excitons	*k*_dec_	10^4^ 1/s	Fit with *J-V* experiment
Pair separation distance	*x*_a_	1.2 nm	Fit with *J-V* experiment
Width of LUMO and HOMO Gaussian	σ_e_, σ_h_	0.128 eV	47–49
Hole mobility	µ_h_	4 × 10^−4^ cm^2^/V.s	50–52
Electron mobility	µ_e_	2 × 10^−3^ cm^2^/V.s	50–52

**Figure 3:**
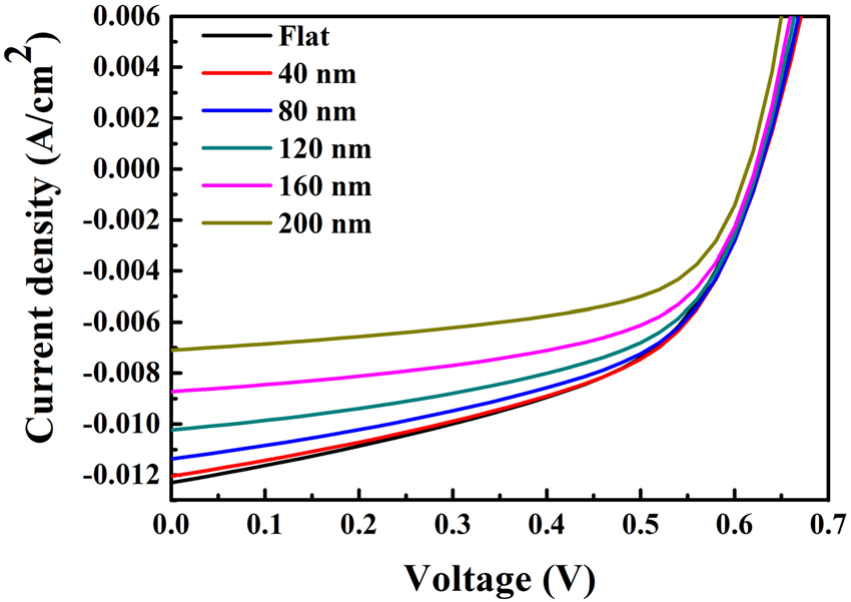
Current density versus voltage (*J-V*) characteristic of the flat OSC, compared to the devices with back contact grating of varying height. For an increased pillar height, *J*_sc_ (in magnitude), *V*_oc_ and PCE decrease. On the other hand, the FF increases with increasing nano-structure height. (Higher *Jsc* compared to Fig. 2 is due to larger active layer thickness (280 nm).

**Figure 4:**
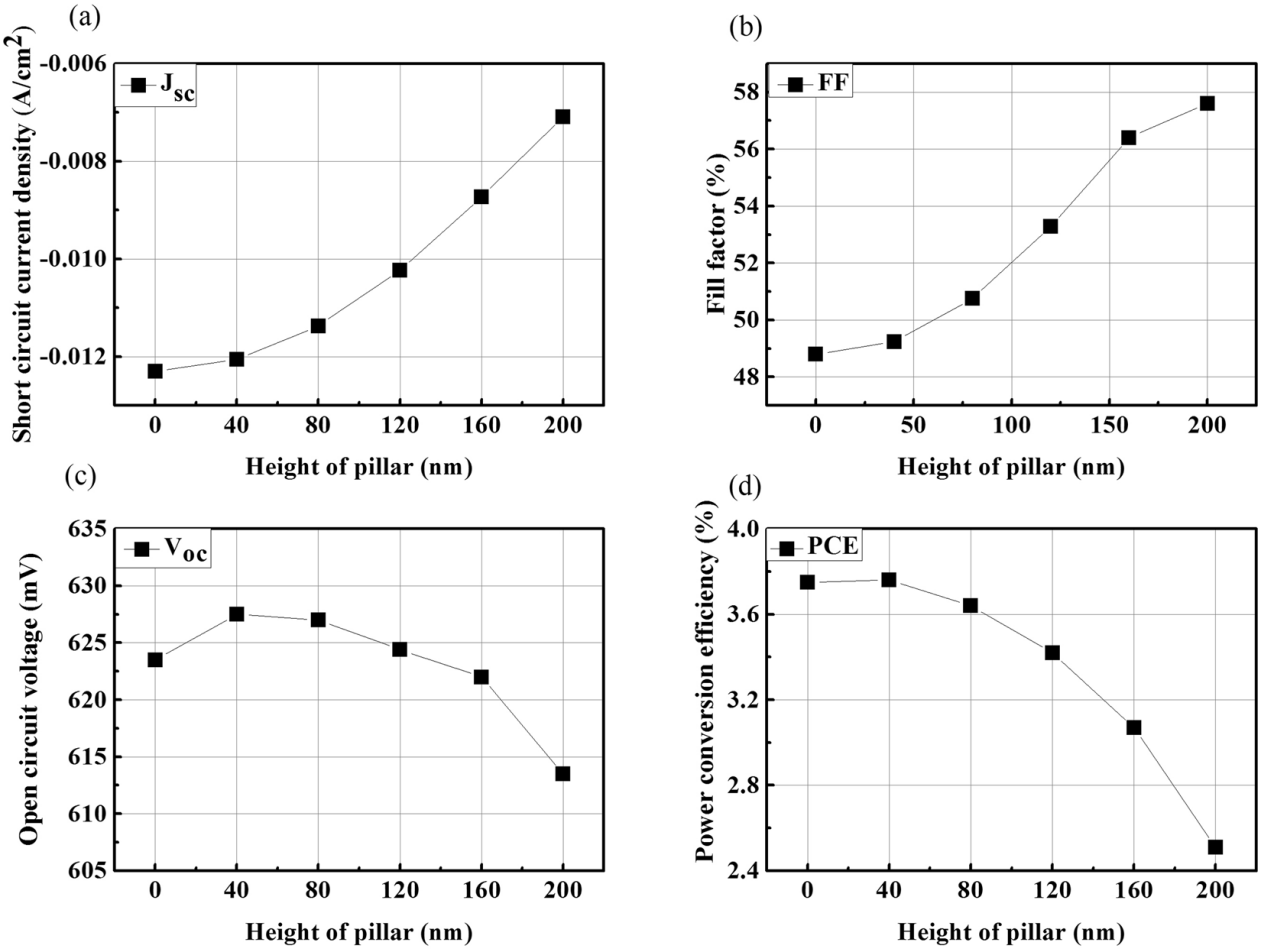
Numerical results for (**a**) the short circuit current density *J*_sc_, (**b**) the fill factor FF, (**c**) the open circuit voltage *V*_oc_, and (**d**) the overall power conversion efficiency PCE of the considered OSC as functions of varying pillar height. While the effect on *V*oc is limited, *J*_sc_ decreases noticeably for higher pillars. This decrease is addressed to the fact that in our simulations the active layer thickness (and not volume) is kept constant, which reduces the active layer volume of the absorbing material with increasing pillar height. However, the PCE reduction is not consistent with the lower absorption, which can be understood by considering the improved collection efficiency and thus, FF enhancement as a function of pillar height.

As a second step, in order to accurately trace the organic solar cell electrical properties, we applied a uniform, constant exciton generation profile in the active layer, allowing for a precise electrical device response analysis. We performed electrical simulations for the modified devices with varying grating geometries, and we compared the results to those of the planar reference structure. We considered a back contact grating architecture (see Fig. 1) with different square pillar heights (*h* = 40, 80, 120, 160 and 200 nm), while keeping the grating pitch (*p* = 400 nm) and the pillar width (*w* = 100 nm) constant. Figure 3 shows the simulated device current density versus voltage (*J-V)* characteristics for different pillar heights. The pillar influence on the open circuit voltage *V*_oc_ is very limited, while the short circuit current density *J*_sc_ is decreasing for taller pillars. This reduction is in line with our expectations, since the active layer height in this simulation is kept constant, and an increased pillar height consequently leads to a reduced effective active layer volume. The reduced active layer volume leads to a lower absorption of light, which, in effect, also decreases *J*_sc_ for increasing pillar heights. This is also the main driver for the decreasing PCE obtained for increasing pillar heights in these simulations. To understand the electrical response of the grating architecture, Fig. 4 depicts the calculated, commonly used, figures of merit, i.e., *V*_oc_, *J*_sc_, fill factor (FF) and PCE, as functions of the pillar height.

**Figure 5:**
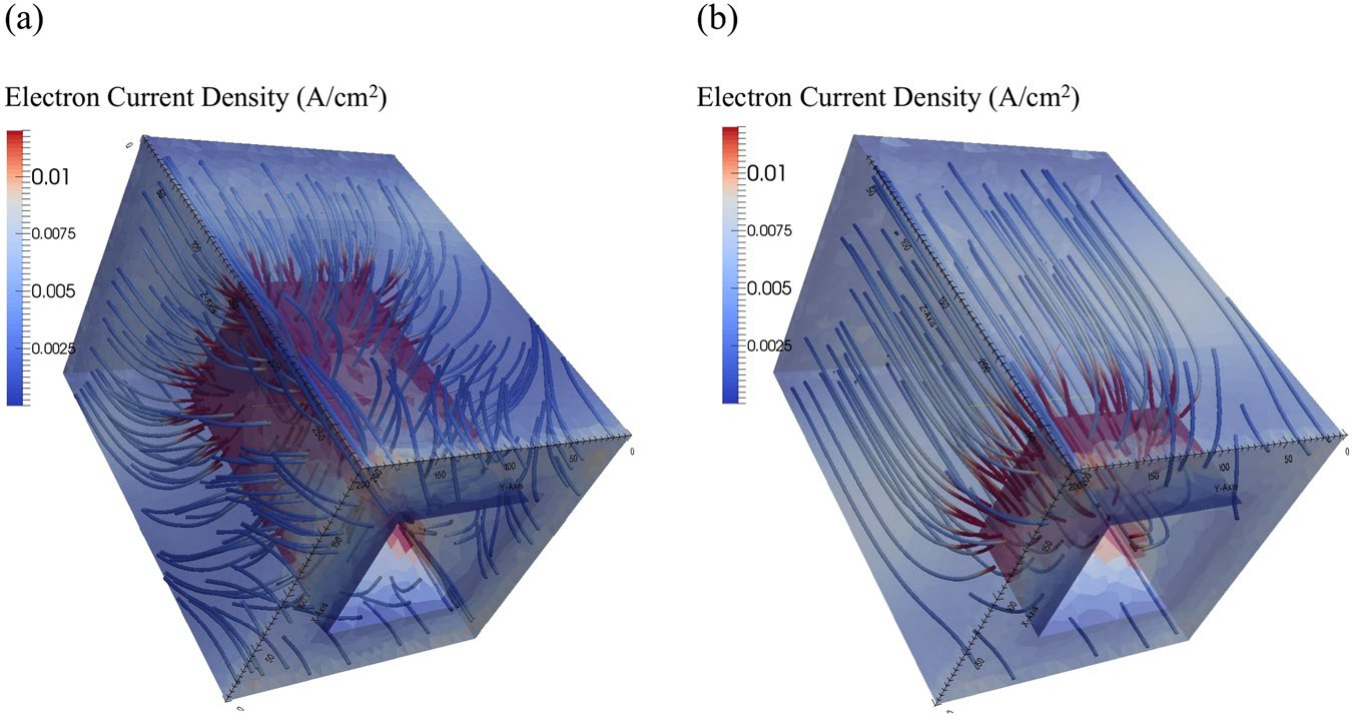
Electron current density magnitude (tubes) within the active layer for two different pillar heights (**a**) h = 200 nm, (**b**) h = 80 nm. By introducing pillars in the organic solar cell, the surface area between the active layer and the back contact increases. This effectively leads to a more efficient free carrier collection and hence to an increased FF as compared to the planar reference cell.

The observed PCE reduction, however, is not consistent with the contribution coming from the lower absorption, and hence *J*_sc_. This observation can be understood by considering the improved collection efficiency, leading to a fill factor (FF) improvement with increased pillar height in the cells. As shown in Fig. 4, a significant FF improvement of 18% can be observed when increasing the grating structure height to 200 nm. This FF improvement is mainly related to an increased interfacial area for collecting charge carriers between the active layer and the back contact, as well as the reduced losses during the free charge carrier transport towards the contact. This FF increase leads to a smaller PCE drop as compared to the one arising solely from the drop in *J*_sc_. Accordingly, as shown in Fig. 5 for the simulated electron collection path and spatial magnitude of the electron current, introducing pillars leads to a more efficient collection of free carriers and hence to a better FF in comparison to the planar cell.

The simulations show that even with an increased FF, the overall PCE in the structured cell is reduced as compared to the planar one (Fig. 4d). This is due to the fact that we use a constant generation profile to examine the electrical behavior of these structures, and thus by introducing the grating structure, the amount of photo-generated carriers is reduced along with the volume of the absorber, which consequently results in lower PCE.

**Figure 6:**
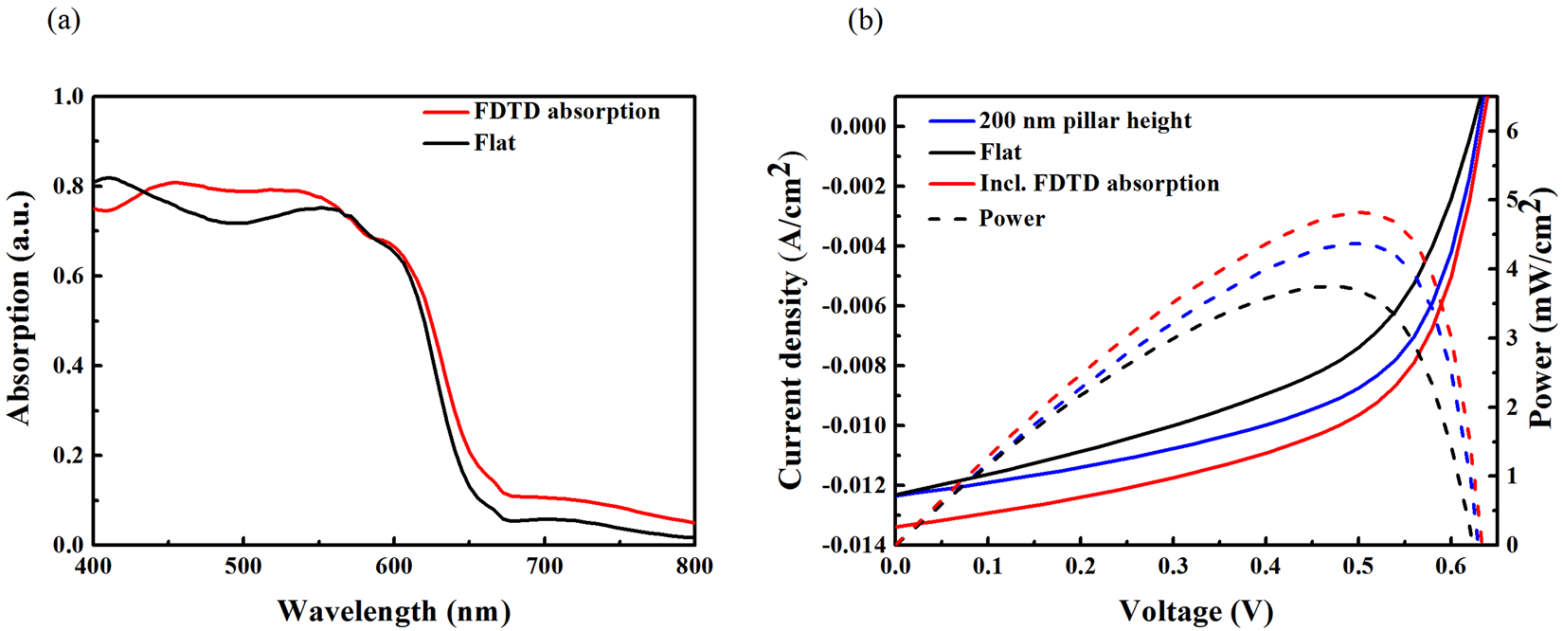
(**a**) Optical absorption of planar and square grating structures with a pillar height of 200 nm. The organic active layer that includes square pillars of height 200 nm shows an 8.8% optical absorption enhancement (averaged over the considered wavelength range). (**b**) Electrical properties of the planar reference solar cell (black line), the grating structure with optical generation profile equalized to the planar cell (blue line) and the solar cell with optical generation profile calculated by the FDTD method (red line). The power conversion efficiency for OSCs having a nano-structured grating enhances, exclusively due to the improved charge collection efficiency. By incorporating the 8.8% optical enhancement within the solar cell, the electrical properties in terms of *J*_sc_, FF and thus PCE (28.5%) improve even further.

In reality, however, several experimental and numerical studies show that even if the integration of nanoparticles or grating structures into the cells reduces the absorber volume, this introduction can still enhance the overall optical absorption efficiency and thus the cell’s *J*_sc_^29–31^. In order to shed light on this discrepancy, finally, we perform optical simulations for same geometries, and subsequently introduce spatially resolved exciton generation rates to our electrical simulator. As shown in Fig. 6a, the optical absorption spectrum calculated for an organic active layer that includes square pillars of height 200 nm shows 8.8% optical absorption enhancement (averaged over the considered wavelength range) compared to a planar solar cell. We note that this is the highest optical absorption enhancement obtained when comparing numerous different pillar heights and widths for 2 different investigated grating pitches. We show the optical absorption enhancement for the different pillar dimensions in Figure S1 in the supplementary information. Figure 6b compares the *J-V* characteristics of the grating-structured cell (blue curve) to the one having a planar geometry (black curve), where the grating structure-influenced exciton generation profile is equalized with the planar one. This shows that the power conversion efficiency for an OSCs comprising a nanostructured grating is enhanced significantly due to improved charge collection efficiency only, i.e., without including the optical (8.8%) enhancement. It should be noted that the PCE could practically improve to higher values through detailed opto-electrical engineering, due to the concurrent optical and electrical improvement offered by this technology. To this end, we simulated the device again, considering the 8.8% optical enhancement (red curve). As demonstrated in Table 2 and by the *J-V* curve (Fig. 6(b)), the PCE increased by up to 28.5%, resulting from both optical absorption enhancement and improved charge collection efficiency. We provide the solar cell performance parameters for different pillar heights, including both the optical absorption and the electrical enhancement, in table S1 in the supplementary information. The maximum *J*_*sc*_ and FF occurs at a pillar height of 200 nm.

**Table 2:** Characteristics of the organic solar cell shown in Fig. 6b.

Device	*V*_oc_ (mV)	*J*_sc_ (mA/cm^2^)	FF (%)	PCE (%)
Planar	623	12.3	48.8	3.74
200 nm pillars	629	12.3	56.5	4.37
200 nm pillars Incl. optical absorption enhancement	629	13.3	57.6	4.82*

^*^Overall PCE improvement (~28.5%) is due to better charge collection efficiency (~18%) and optical enhancement (~8.8%).

In general, our approach is applicable to several complex geometries and material systems. By comparing the results obtained with experimental measurements of similar structures, the calculated absorption and FF enhancement can be qualitatively validated^31–35^. For example, for similar structures to those studied here, it has been shown experimentally in ref. 30 that cell performance and FF is enhanced (25.5% and 19.4%, respectively) by nano-patterned PEDOT:PSS, using nano-imprint lithography, which can be explained qualitatively with our analogous simulation study.

## Conclusion

In this work we have studied the electrical properties of three-dimensional organic solar cells with a back contact grating structure. We have demonstrated that the cells structured with square pillars can lead to a 18% fill factor enhancement as compared to the planar OSC. We address this fill factor enhancement to an increased interfacial area for collecting charge carriers, which results in reduced losses and improved charge collection efficiency. The highest fill factor for the studied structure is found when the grating height is at the tested maximum of 200 nm, which is achievable with several available lithography techniques. In addition, optical simulations show an 8.8% absorption enhancement inside the active layer. Our results confirm that, by implementing nanostructured gratings in organic solar cells, beyond optical enhancement, we are able to increase the power conversion efficiency of solar cells due to a concurrent enhancement of both the electrical and optical cell characteristics.

## Methods

### Electrical simulation

For exciton dynamics and free charge transport, we developed a comprehensive numerical model, based on the drift-diffusion approximation and a variant of Koster *et al*.’s^36^ concept of exciton continuity. Our model includes several fundamental physical processes specifically associated to organic semiconductors that allow us to consider the effect of energetic disorder and electrode properties. The developed model (a detailed mathematical description can be found in ref. 28) overcomes most of the common approximations previously used in simulating the opto-electrical properties of OSCs. One important feature of our approach is that we apply a reliable model at the organic-metal interfaces, using a combination of the Mott-Schottky and the Scott-Malliaras model. Furthermore, we consider the fundamental difference between carrier transport in inorganic semiconductors (which assume the presence of a well-defined energy band edge) and in organic semiconductors (which include a hopping mobility model and a Gaussian density of states model). Such a modification is significant for a reliable approximation of organic semiconductors, which consist of disordered polymeric or molecular structures bonded by weak Van der Waals interactions. The main simplification in our approach is that the model treats the active layer as a single bulk hetero-junction (BHJ) material, considering an effective medium approximation, where the real interfaces between donor and acceptor are not explicitly considered. Since the simulation of realistic donor/acceptor blend morphology is computationally expensive and requires several input parameters that cannot be derived by measurements, this simplification is common in the drift-diffusion approach for BHJ layer modeling.

### Optical Simulation

We performed the optical analysis using the FDTD method, employing the commercially available *FDTD Solutions* software package from Lumerical to calculate the spatial electromagnetic field distribution, versus time and position, in a 3D domain. We employed periodic (Bloch) boundary conditions for the lateral (*x*- and *y*-) directions, and perfectly matched layer (PML) boundary conditions at the contacts in the vertical (*z*-) direction. As illumination source, we used a normally incident plane wave polarized along the *x*-axis. In order to address the plasmonic behavior correctly, we chose a spatial mesh size of 4 nm in the regions surrounding the nanostructures. We chose the material refractive indices according to the literature^37–40^. We normalized the results to the air mass (AM) 1.5 spectrum. Based on the calculated electric field intensity profile at each incident wavelength inside the organic active layer, the number of photons absorbed can be calculated by

1 $$G(\lambda )=\frac{{|E(\lambda )|}^{2}imag(\varepsilon )}{2\hslash }$$

where the *E* is electric field, *ε* is a electric permittivity, λ denotes the wavelength and $\hslash$ is the reduced Planck constant. Assuming that each absorbed photon generates an exciton in OSCs, we subsequently calculate the exciton generation rate inside the organic active layer, by integrating *G (λ*) over the incident light spectrum^39, 41^.

## Electronic supplementary material

The influence of electrical effects on device performance of organic solar cells with nano-structured electrodes
