# Supplementary material for: The influence of electrical effects on device performance of organic solar cells with nano-structured electrodes
Source: Sci Rep. 2017 Jul 13;7:5300. doi: 10.1038/s41598-017-05591-8 (PMC5509690; doi:10.1038/s41598-017-05591-8)
Supplement: Supplementary file 1 — The influence of electrical effects on device performance of organic solar cells with nano-structured electrodes [file 41598_2017_5591_MOESM1_ESM.pdf]

The influence of electrical effects on device performance of organic solar cells with nano-structured electrodes

Mina Mirsafaei[1], Amir Hossein Fallahpour[2], Paolo Lugli[2], Horst-Günter Rubahn[1], Jost Adam[1] and Morten Madsen[1]

[1]SDU NanoSYD, Mads Clausen Institute, University of Southern Denmark, Alsion 2, Sønderborg, DK-6400, Denmark;

[2] Department of Electrical and Computer Engineering, Institute for Nanoelectronics, Technical University of Munich, Arcisstr. 21 80333, Munich, Germany;

We performed numerical simulations to calculate the optical enhancement, in comparison to the planar solar cells, by varying the pillar height and width. We found a maximum of 8.8% optical absorption enhancement for pillars with height h=200 nm, width w= 100 nm and pitch p=400 nm. Figure S1 shows the optical absorption enhancement for different grating structure geometries.

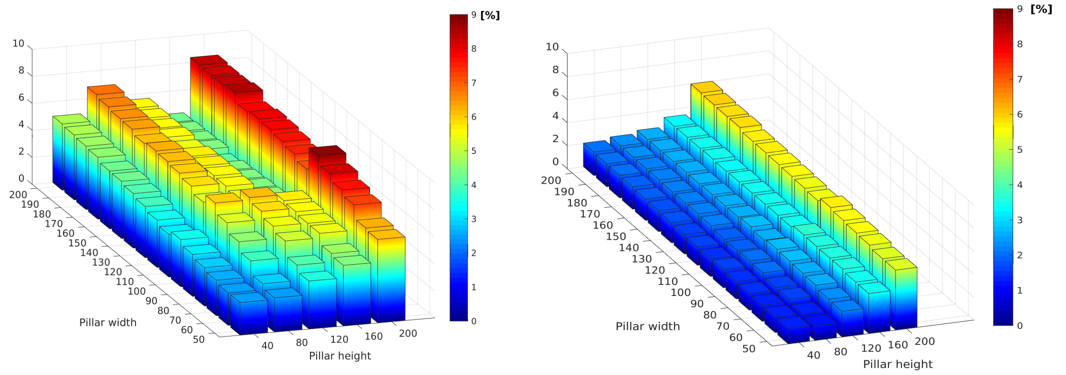

Figure S1: Optical enhancement for varying pillar widths and heights and a grating pitch of 400 nm (left) and 800 nm (right). The maximum 8.8% optical absorption enhancement is achieved at h=200 nm, w=100 nm and p=400 nm. These optical numerical simulations demonstrate that the optical absorption enhancement is higher for a 400 nm grating pitch compared to a 800 nm grating pitch.

Following our findings on the optically optimized structure, we performed the electrical simulations, including the optical absorption enhancement, for varying pillar heights, at w=100 nm and p=400 nm. We find that the organic solar cell fill factor (FF) increases with increasing pillar height, due to the increased interfacial area for collecting charge carriers. The maximum FF enhancement is obtained for a pillar height of 200 nm (Table S1).

Table S1: Solar cell performance for different pillar heights including the optical absorption enhancement.

Device	V_{oc} (mV)	J_{sc}(mA/cm^2)	FF (%)	PCE (%)
Planar	623	12.3	48.8	3.74
40 nm	627	13.18	49.24	4.07
80 nm	627	13.24	50.75	4.2
120 nm	624	12.77	53.29	4.24
160 nm	622	12.61	56.4	4.42
200 nm	629	13.3	57.6	4.82
